# Developments in the health situation in Germany during the initial stage of the COVID-19 pandemic for selected indicators of GEDA 2019/2020-EHIS

**DOI:** 10.25646/7172.2

**Published:** 2020-12-09

**Authors:** Stefan Damerow, Alexander Rommel, Franziska Prütz, Ann-Kristin Beyer, Ulfert Hapke, Anja Schienkiewitz, Anne Starker, Almut Richter, Jens Baumert, Judith Fuchs, Beate Gaertner, Stephan Müters, Johannes Lemcke, Jennifer Allen

**Affiliations:** Robert Koch Institute, Berlin Department of Epidemiology and Health Monitoring

**Keywords:** SARS-COV-2, MENTAL HEALTH, BMI, SMOKING, HEALTH CARE UTILISATION, ASSISTANCE AT HOME

## Abstract

SARS-CoV-2, the novel coronavirus, has posed major challenges in Germany in 2020. It is unclear whether the pandemic and containment measures will have an impact on the health of the population beyond the point of infection. The German Health Update (GEDA 2019/2020-EHIS) is a nationwide survey of the population aged 15 years and older (n=23,001) that was conducted between April 2019 and September 2020. The focus of the analysis was on indicators for which pandemic-related changes could be expected. Based on regression models, adjusted proportions and mean values were estimated as trends over time. Any differences in the values found for the time period of containment measures in spring 2020 and the reference period 2019 were statistically tested. Since the implementation of containment measures, both body weight and body mass index (BMI) have increased. The utilisation of general and specialist medical services decreased temporarily. The number of tobacco smokers during the observation period also decreased, yet without revealing a clear link to the pandemic situation. No differences were found in the general population for depressive symptoms and household assistance received and provided. During the period of containment measures, changes to the health situation beyond the occurrence of infections can be observed. However, a more differentiated explanation of these findings will require further analyses.

## 1. Introduction

Since the beginning of 2020, the novel coronavirus SARS-CoV-2 (Severe Acute Respiratory Syndrome Coronavirus 2) has been spreading globally at enormous speed creating major challenges even in Germany. With the aim of containing the spread of the virus, a few German federal states implemented social distancing measures and bans on large events as early as the beginning of March. From mid to end March, the Federal Government decided on extensive measures to contain the spread of the pandemic (partial lockdown) which were coordinated with the federal states and then gradually eased again from the end of April onwards. During this period, schools, most shops, all gastronomy services, many businesses and public institutions were closed and strict social distancing measures were imposed in public areas. Large events or gatherings and any kind of celebrations were prohibited.

**GEDA 2019/2020-EHIS**

Fifth follow-up survey of the German Health Update

**Data holder:** Robert Koch Institute

**Objectives:** Provision of reliable information on the health status, health behaviour and health care of the population living in Germany, with the possibility of European comparisons

**Study design:** Cross-sectional telephone survey

**Population:** German-speaking population aged 15 and older living in private households that can be reached via landline or mobile phone

**Sampling:** Random sample of landline and mobile telephone numbers (dual-frame method) from the ADM sampling system (Arbeitskreis Deutscher Markt- und Sozialforschungsinstitute e.V.)

**Sample size:** 23,001 respondents

**Study period:** April 2019 to September 2020

**GEDA survey waves:**

- GEDA 2009
- GEDA 2010
- GEDA 2012
- GEDA 2014/2015-EHIS
- GEDA 2019/2020-EHIS

**Further information in German is available at** www.geda-studie.de

There is a fear that, in addition to people contracting the disease and becoming ill, the containment measures would entail detrimental secondary effects, i.e., the cancelling of doctors’ appointments or social isolation [1]. While initial reviews did indeed show a decline in inpatient treatment, an increase in the utilisation of outpatient tele-medical care services was also observed [1]. A spike in mental disorders, i.e. depression, adjustment problems, anxiety disorders or trauma sequelae was also a concern [2, 3]. Containment measures and their effect on changes in everyday life may also have had an impact on people’s dietary habits and physical activity patterns, and this could potentially also have affected body weight [4]. In addition, in the initial stage of the pandemic, smoking was discussed as a risk factor for a severe course of COVID-19 disease [5], with increased attempts to quit smoking as a plausible consequence. At the same time, smokers experience smoking as a stress-relieving factor, possibly leading them to increase their consumption of tobacco products [6]. For households with smokers, it is conceivable that non-smoking household members were exposed to more passive smoking during COVID-19 containment measures [7]. In addition, against the backdrop of health inequalities in the population, there were discussions as to whether socially disadvantaged population groups have been more affected by the burdens associated with infection control measures [8] and whether, for example, changes to health behaviour relate to social factors [4].

The German Health Uptate (GEDA 2019/2020-EHIS) [9] allows to analyse changes in health status, health behaviour and utilisation of medical services during the beginning of the pandemic. The study started in April 2019, approximately one year before measures to contain the pandemic began in Germany, and ended in September 2020, after most bans had been lifted and restrictions had been significantly eased. With over 20,000 participants, analyses at individual sections of the study period for a selection of health indicators are possible. This paper focuses on a selected set of analyses for the developments over time in the areas of mental health, health behaviour, utilisation of medical services and assistance at home. We also look at whether there have been different developments for women and men or in individual age and education groups during the observation period.

## 2. Methodology

### 2.1 Study design, sampling and weighting

#### Study design

GEDA 2019/2020-EHIS is a nationwide cross-sectional survey of the population aged 15 and older living in Germany. On behalf of the Federal Ministry of Health, the Robert Koch Institute (RKI) has conducted the GEDA study at intervals of several years since 2008 and the survey is part of RKI health monitoring [10, 11]. As in the 2014/2015 wave, the questionnaire of the European Health Interview Survey (EHIS) was fully integrated, supplemented by additional questions and extended to cover the resident population aged 15 years and older [12, 13]. The most recent GEDA wave was conducted as a telephone interview survey using a computer assisted, fully structured interview (i.e. Computer Assisted Telephone Interview, CATI). It was based on a random sample of landline and mobile telephone numbers. The population comprises residents in Germany aged 15 years and older living in private households who usually reside in Germany at the point of data collection. Sampling was conducted based on the telephone random sampling system of the ADM (Arbeitskreis Deutscher Markt- und Sozialforschungsinstitute e.V.). This system is based on a ‘dual-frame’ approach, in which two subsets (mobile and landline numbers) are used [14]. This method allows for an (almost) complete coverage of the population. The data were collected by interviewers from a market and social research institute. The Robert Koch Institute accompanied the entire survey process through continuous supervision and implementation of quality assurance measures.

#### Sample

**Annex Figure 1:**
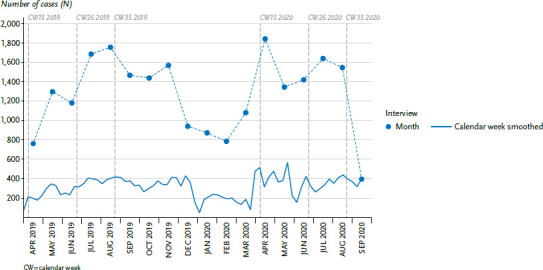
Development over time of the interviews carried out in GEDA 2019/2020-EHIS Source: GEDA 2019/2020-EHIS

The survey took place between April 2019 and September 2020. A total of 23,001 people (12,111 women, 10,890 men) with complete interviews participated in the GEDA 2019/2020-EHIS study. Based on the standards of the American Association for Public Opinion Research (AAPOR), the response rate was 21.6% (RR3)[15]. On average, 1,278 people (minimum: 394 persons, maximum: 1,841 persons) took part in the survey each month. The average number of interviews per calendar week was 304 persons (minimum: 46 persons, maximum: 564 persons; Annex Figure 1). The calendar weeks 15 to 26 and 15 to 35 in 2019 and 2020 included 7,312 study participants (2019: 3,117 persons, 2020: 4,195 persons) and 14,100 study participants (2019: 6,613 persons, 2020: 7,487 persons).

#### Weighting

For data weighting, design weighting was first applied to account for the different selection probabilities (of mobile and landline numbers). A standard dual-frame design calculation method was used. Subsequently, an adjustment based on the official population figures was carried out with regard to age, sex, federal state and district type (as at 31 December 2018). In addition, weighting also accounted for the distribution of education levels according to the International Standard Classification of Education (ISCED classification) in the microcensus (2018). Containment measures, e.g. recommendations to work from home or social distancing measures, potentially affected the participation of specific population subsets, such as the working population. For this reason, the sample before and from the cut-off date 16 March 2020 (adoption of the agreement between the federal government and federal states on guidelines to slow down the spread of the coronavirus) was adjusted separately with the marginal distributions for age, sex and education.

### 2.2 Indicators

The selection of topics focused on health monitoring indicators that could have been expected to change as a result of measures to contain the COVID-19 pandemic. Based on the available literature (see Chapter 1 Introduction), effects in health status (especially mental health), health behaviour, health care and support services were assumed to have potentially occurred. Methodologically, only those indicators were considered which explicitly aimed to record facts at the time of the survey (e.g. ‘currently’). Indicators related to longer periods of time (e.g. ‘in the last twelve months’) were not considered capable of adequately capture possible consequences of the containment measures.

#### Mental health

Depressive symptoms were surveyed based on the self-reported data provided by participants according to the internationally established 8-item Patient Health Questionnaire (PHQ-8) [16]. This instrument evaluates symptoms of major depressive disorder in accordance with the criteria established by the Diagnostic and Statistical Manual of Mental Disorders (DSM-IV, 4th edition [17]) with regard to their occurrence within the last two weeks. Depressive symptoms are defined as a score of at least ten out of a maximum of 24 points.

#### Body weight and body mass index

Body weight and height data are based on the information provided by respondents. Body height is surveyed by the question: ‘How tall are you when you are not wearing shoes?’ The information was given in centimetres. Body weight was recorded by asking: ‘How much do you weigh without clothes and shoes? Please state your body weight in kilograms’. The Body Mass Index (BMI) is then calculated as the ratio between body weight and height square (kg/m^2^).

#### Tobacco smoking and passive smoke exposure

Smoking status was surveyed by asking: ‘Do you smoke tobacco products, including use of tobacco heaters? Please exclude electronic cigarettes or similar products.’ (answer categories: ‘yes, daily’, ‘yes, occasionally’, ‘no, not anymore’, ‘I have never smoked’). On the basis of these response categories a dichotomous variable is formed in the present paper, which distinguishes between current smokers (daily or occasional) and current non-smokers (former smokers or non-smokers). The use of electronic cigarettes or similar electronic products was not the subject of the present analysis. Data on exposure to passive smoking was collected by asking the following question: ‘How often are you exposed to tobacco smoke indoors? By indoors we mean, for example: at home, at work, in public buildings or in a restaurant’. Using the answers provided, the daily degree of exposure to passive smoking is presented as a dichotomous variable for current non-smokers. Daily exposure to passive smoking was defined as comprising either ‘daily, 1 hour or more’ or ‘daily, less than 1 hour’ exposure to passive smoking.

#### Utilisation of medical services

Medical services utilisation was recorded with the question: ‘How often in the last 4 weeks have you consulted a general practitioner or family doctor for advice, examination or treatment?’ Visits to a medical specialist were recorded using the same wording. Two dichotomous variables were formed to distinguish respondents who had consulted a general practitioner (GP) or a specialist from those who had not.

#### Assistance received and provided

To identify those requiring support in the household, people aged 55 years and older (n=12,054) were first asked about difficulties in carrying out various household activities. Based on an established measurement of instrumental activities of daily living [18], the following activities were assessed: (1) preparing meals, (2) using the telephone, (3) shopping, (4) managing medication, (5) doing light housework, (6) occasionally doing heavy housework, and (7) taking care of finances and everyday administrative tasks. People who had difficulties in at least one activity were then asked about support they had received: ‘Now think of all the household activities you have difficulty doing without help. Do you usually have help with any of these activities?’ (Answer categories: ‘Yes, with at least one activity’ versus ‘No’), resulting in a dichotomous variable ‘assistance received’ (yes versus no). In order to capture a lack of necessary support, (a) persons who received support were asked whether they needed more help with at least one of the activities and (b) people who received no support, whether they needed help. On this basis, the variable ‘lack of support’ (yes versus no) was created.

Provided assistance or care giving was recorded by asking the following question: ‘Do you provide care or assistance to one or more persons suffering from some age problem, chronic health condition or infirmity, at least once a week? Exclude any care provided as part of your profession’. A dichotomous variable ‘assistance provided’ (yes versus no) was generated.

#### Education

Educational levels according to the CASMIN classification (Comparative Analyses of Social Mobility in Industrial Nations) were used as an indicator of social status. School and Vocational Education and Training (VET) qualifications served to distinguish three groups with low, medium and high education [19].

### 2.3 Statistical analyses

Applying the weighting factors, three logistic regression models were estimated for dichotomous indicators and three linear regression models for metric indicators. Federal state, age, sex, education as well as the interactions between age, sex and education were used as independent control variables. A detailed description of all models is provided in the annex (Annex Table 1).

To show the developments for indicators over the survey period, the first model used the interview month as an independent, categorical variable. To smooth out model 1 figures, a trend over time was modelled for the second model using a polynomial of degree four for the interview week. The results of the two model estimates were used to calculate adjusted predictions stratified by interview month (model 1) and interview week (model 2). For the dichotomous indicators, the predictions can be interpreted as adjusted proportions (in %) and for the metric indicators as adjusted mean values. The results are presented in a graph, including a 95% confidence interval for each indicator.

In addition to the graphical representation, the periods between calendar weeks 15 and 26 in 2019 and 2020 were compared to reveal potential impacts on the indicators due to the pandemic situation in the spring of 2020 compared to 2019 values. As data collection only started in April 2019, the calendar weeks of March cannot be included in the comparison. The potential effects for the health indicators body weight and BMI can be expected to occur with a certain time lag, the period of comparison for these indicators was therefore extended to cover calendar weeks 15 to 35. To test the statistical significance of the comparisons for these periods from 2019 and 2020, the sample was restricted to interviews from the defined periods and a regression model was estimated in each case, which used a binary variable to differentiate the periods instead of the interview month or the interview week (model 3). A statistically significant difference between the time periods is assumed if the p-value of the binary variable is less than 0.05. Furthermore, the result of the model estimation is used to calculate adjusted proportions or adjusted mean values for the periods (Annex Table 2). To evaluate differentiated developments between the periods with regard to sociodemographic variables, interactions with age, sex and educational groups were tested. All analyses were conducted with StataSE 15.1 software (Stata Corp., College Station, TX, USA, 2017).

## 3. Results

### 3.1 Mental health

**Figure 1:**
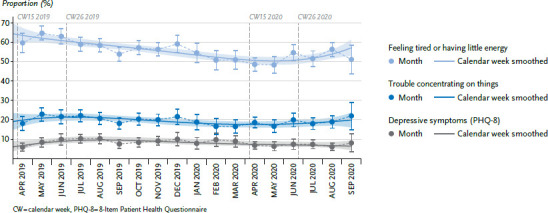
Mental health over time, from April 2019 - September 2020 (adjusted proportions) Source: GEDA 2019/2020-EHIS

Throughout the entire observation period, the adjusted proportions of persons with depressive symptoms have remained relatively constant with no conspicuous changes from spring 2020 onwards (Figure 1). In the period between calendar week 15 and calendar week 26, the value was 6.6% in 2020 and 8.3% in 2019. Two items of PHQ-8 had a lower value between calendar weeks 15 and 26 in 2020 than in the reference period in 2019: ‘Feeling tired or having little energy’ decreased from 64.0% to 50.7% and ‘Trouble concentrating on things, such as reading the newspaper or watching television’ decreased from 21.9% to 18.1% (Annex Table 2). However, the time course of the two items does not indicate that the decrease is due to any relevant change from March 2020 onwards (Figure 1).

### 3.2 Body weight and Body Mass Index

**Figure 2:**
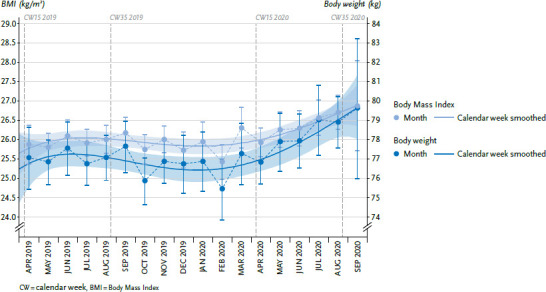
Body weight and BMI over time, from April 2019 - September 2020 (adjusted mean values) Source: GEDA 2019/2020-EHIS

Fluctuations in estimated mean BMI and body weight are observed over the entire observation period. From spring 2020 onwards, there is a clear increase (Figure 2). In the period from April to August 2019, the adjusted mean body weight was 77.1 kg. In the reference period from April to August 2020, this value was 78.2 kg. This shows an increase by about one kilogram between corresponding months in 2019 and 2020. This difference is statistically significant. BMI also increases: in the period from April to August 2020, the adjusted average BMI of 26.4 kg/m^2^ was higher than the adjusted average BMI of 25.9 kg/m^2^ in the period from April to August 2019 (Annex Table 2).

### 3.3 Tobacco smoking and passive smoking

**Figure 3:**
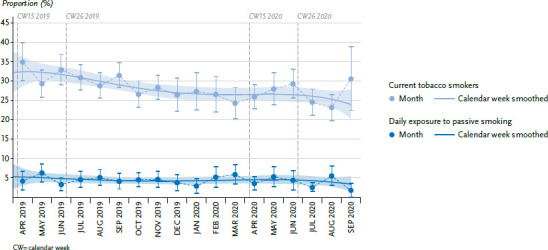
Tobacco smoking and passive smoking exposure over time, from April 2019 - September 2020 (adjusted proportions) Source: GEDA 2019/2020-EHIS

The estimated proportions of tobacco smokers fluctuated slightly with an overall slight decrease during the survey period of the GEDA 2019/2020-EHIS study between April 2019 to September 2020. No change is observed for the phase of containment measures (Figure 3). However, when comparing the period between calendar weeks 15 and 26 in 2019 with the same weeks the following year, the adjusted tobacco smoking rate decreased from 32.6% to 28.1%. This time comparison does not show any changes in daily exposure to passive smoking. In both 2019 and 2020, the proportion of people exposed to passive smoking in the population during the period in question was estimated at five percent (Annex Table 2).

### 3.4 Medical services utilisation

**Figure 4:**
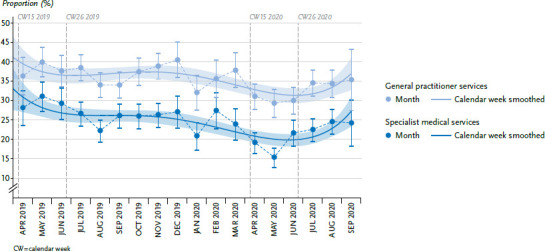
Outpatient medical services utilisation over time, from April 2019 - September 2020 (adjusted proportions) Source: GEDA 2019/2020-EHIS

Outpatient GP and specialist medical services utilisation by the population is subject to considerable seasonal fluctuations. Containment measures led to a marked decline in the utilisation of outpatient GP and specialist services between calendar weeks 15 and 26 in 2020, to a level below the seasonal lows of August 2019 and January 2020. This decline is visible for GP services from April and in March for specialist services (Figure 4). The statistical test comparing the values from the reference periods of 2019 and 2020 is statistically significant. Between calendar weeks 15 and 26 2019, the uptake of GP medical services was 38.4% and 29.7% in the same period in 2020. The utilisation of specialist services declined from 30.0% in 2019 to 17.7% in 2020. From July 2020, the utilisation of medical services begins to increase again and returns to comparable levels as in the reference months of 2019 (Annex Table 2).

### 3.5 Assistance received and provided

**Figure 5:**
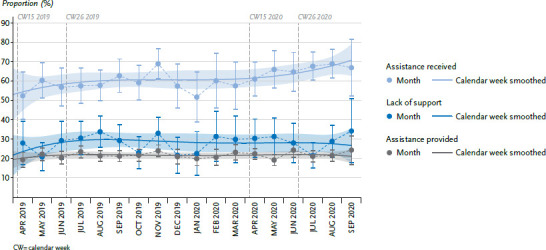
Assistance received and provided over time, from April 2019 - September 2020 (adjusted proportions) Source: GEDA 2019/2020-EHIS

In Figure 5, the course of the three curves shows fluctuations in the respective proportions over the entire observation period and no changes that could be attributed to the pandemic situation in spring 2020 are apparent. In a comparison between 2019 and 2020 of calendar weeks 15 to 26, the adjusted proportion of persons receiving support with household activities increased slightly from 56.5% to 61.8%. The adjusted proportion of respondents who considered that they needed more help with at least one activity also increased slightly from 26.2% to 29.1%. Both increases are not statistically significant (Annex Table 2). If these results are considered separately according to age groups, the results reveal a strong increase in the proportion of people in the 55-to 64-year-old age group who indicate they might need more help; in contrast, this proportion decreased significantly for people in the over 80 age group. A comparison between calendar weeks 15 to 26 in 2019 and 2020 shows no significant difference in the adjusted proportions of persons providing care or assistance (2019: 20.7% and 2020: 22.0%) (Annex Table 2).

### 3.6 Differences according to age, sex and education

The statistical tests carried out to compare calendar weeks 15 to 26 and 15 to 35 of the years 2019 and 2020 for differences in health outcomes according to age, sex and education show few noteworthy or significant results. The trends found do not differ systematically for women or men, or for education groups. One exception is GP and specialist services utilisation. Here, significant differences by education group can be seen insofar as utilisation in the high and low education group decreases more strongly than in the medium educational group. Furthermore, a stronger increase in a lack of support with household activities can be observed among 55-to 64-year-olds.

## 4. Discussion

For the period of containment measures, changes to health situation beyond contracting the infection can be observed. Graphical analyses and comparisons for the values from the periods in 2019 and 2020 show an increase for body weight and BMI and a temporarily strong decrease in the use of general and specialist medical services. The proportion of tobacco smokers in the population has also fallen. However, a direct link to the pandemic situation in spring 2020 is unclear. No noticeable differences were found for the general population regarding depressive symptoms and assistance received and provided.

One of the limitations of telephone surveys is that the length of interviews can have an influence on data quality [20]. Since it is more susceptible to social desirability, the “true” prevalence of potentially sensitive items can be underestimated [21]. In addition, response rates are generally lower than in face-to-face interviews, which, however, does not necessarily imply a higher non-response bias [22]. The present results assume that the sample does not show systematic bias due to the containment measures. Possible factors have already been considered by weighting according to age, sex and education. Moreover, initial analyses do not show a systematic selection between the sub-samples of the comparison periods 2019 and 2020, but it cannot be completely ruled out that a change in willingness to participate has had an impact on certain health indicators. For example, the implementation of short-time work or the expansion of flexible homeworking may have made it easier or harder to reach specific population groups by phone. In-depth methodological analyses will need to clarify whether weighting effectively offset such factors. Due to these limitations, this paper so far does not report evaluations for specific risk groups. Moreover, the number of cases for such groups is often not sufficiently high to statistically verify possible differences over time.

At the population level, initial fears that mental disorders could increase as a result of the COVID-19 pandemic or the containment measures are not initially supported by the available results. No changes were found for depressive symptoms during strict containment measures or when restrictions were eased again. There was even a downward trend as regards two of the depressive symptoms, a feeling of tiredness or having little energy, and having difficulty concentrating. Tiredness, loss of energy and difficulty concentrating are classic symptoms of occupational stress [23]. On the other hand, it is clear that this decline follows a continuous trend and should therefore not be seen as a positive effect of the containment measures. Although the present study does not provide analyses comprehensively for all mental disorders, depressive symptoms do not only indicate depression but occur in numerous other mental disorders. These findings are in line with an analysis by the Central Institute for Mental Health, which also used a random sample of the German population (n=721) in April 2020 and found no changes in the frequency of mental health symptomatology compared to 2018 [24]. Data from the Netherlands (n=3,983) also indicated that the levels of anxiety and depressive symptoms did not change compared to the previous year [25]. It remains to be seen how the mental health outcomes play out in the general population because they will hinge on the on-going course of the pandemic, the measures taken and the possible economic and social consequences. It cannot be predicted to what extent the population will continue to be resilient whether prevention measures and healthcare will be sufficient or whether the previously expressed fears, including increased suicide rates, will actually materialise [26]. International findings do indicate that higher COVID-19 incidence rates and stricter measures can increase the burdens on mental health, which could then also lead to an increase in mental disorders [27].

The analyses presented here do seem to confirm previous findings on body weight and BMI developments. Measures to contain the COVID-19 pandemic would then have led to changes in daily life that could have caused people to gain weight. Two further surveys provide such findings. However, the methodological quality of these surveys is highly heterogeneous and, as they do not claim to be representative, they have their limitations. In the nu3 Corona study carried out online on 22 and 26 April 2020, 24% of respondents said that they had gained weight since containment measures were introduced in mid-March. More than one in two respondents said they had gained between one and three kilograms [28]. In the YouGov online survey conducted in mid-May, 14% of respondents reported that they had gained between one and two kilos during the containment measures, and 12% between two and five kilograms [29]. Reasons for weight gain were eating more often and unhealthy foods as well as doing less exercise. Gaining one kilogram does not appear to be relevant at an individual level and this dimension is not clinically significant. However, a longitudinal analysis of cohort studies that were conducted in Germany between 1994 and 2007 showed that on average men in the 45-to 64-year-old group in Germany annually gain only 250g and women 240g [30]. Whether body weight and BMI continue to rise across the population over the next few months should be further monitored.

The available data showed no unusual development in the frequency of tobacco smoking or exposure to passive smoking. The decrease in tobacco smoking prevalence seems plausible in view of the long-term decrease seen for smoking [31]. As other studies have shown, both a decrease and an increase in the proportion of tobacco smokers and passive smoke exposure would have seemed plausible during the phase of containment measures [32, 33]. Whether the changes in smoking behaviour are a direct consequence of the COVID-19 pandemic containment measures cannot be assessed on the basis of the available data.

The developments for the utilisation of outpatient medical services during the survey period seem plausible. Seasonally low utilisation rates are observed primarily during the holiday months (summer of 2019 and at the beginning of 2020). Then containment measures caused medical services utilisation rates to actually fall below holiday time levels. Although basic medical care was maintained during this period, the population seems to have increasingly refrained from using outpatient medical services. In Germany, the main focus of research has so far been on changes in the use of emergency medical services, with a sharp decline being observed here too [34–36]. The same applies to hospital care utilisation [37, 38]. This is in line with other national and international findings whereby social distancing measures have led to a significant decline in the utilisation of dental and psychiatric emergency care or the use of imaging procedures for example [39–41]. The extent to which the quality of medical care has suffered as a result of dispensing with medically necessary treatments cannot be answered on this basis.

For the population aged 55 years and older, who experience difficulties with everyday household activities, the results show that sufficient assistance during the time of containment measures due to the COVID-19 pandemic. Obviously family and neighbourhood networks or even professional support were available and used to a sufficient extent. Only among people aged 55 to 64 years a lack of assistance was found. This is at the same time the age group that most frequently provided assistance and care. It should be considered that people aged 55 to 64 years, provide support to older and very old parents, for example, apparently do themselves not receive enough help or haven't yet been able to organise sufficient support. Due to multiple burdens, this group could be susceptible to health consequences and does not seem to have received sufficient attention so far [42].

GEDA 2019/2020-EHIS allows to evaluate indicators of health over time and to systematically compare changes in 2020 during the phase of measures to contain the COVID-19 pandemic, with the same period of the previous year. This comparison did not yield a uniform picture. In some areas, such as mental health, the feared increase of depressive symptoms and lack of support for household activities were not confirmed. In other areas, there have been systematic shifts for the period of containment measures and these should be further investigated. Increases to body weight and BMI highlights indicators that should be monitored in the longer term. Another example is depressiveness. While no changes are initially apparent, it should be noted that, regarding the COVID-19 pandemic, individual population groups faced very different challenges and burdens. Future research should therefore examine whether specific developments that were not the focus of this study can be identified for certain population groups, for example people on low incomes, the unemployed, single parents, the elderly or people with chronic diseases. For example, the development of the utilisation of medical services for elderly or chronically ill persons should be analysed more closely. Continuing GEDA 2019/2020-EHIS beyond the originally planned period (end of 2020) will offer opportunities for more in-depth analyses as well as for the observation of longer-term developments. A longer study period will make it possible to further substantiate the investigated findings and to carry out more in-depth evaluations over and above the topics presented here.

## Key statements

- Almost no changes in frequency of depressive symptoms were observed during the measures to contain the COVID-19 pandemic.
- Average body weight and Body Mass Index were higher in the 2020 observation period than in the same period of the previous year.
- The proportion of tobacco smokers dropped during the period of containment measures compared to the same period last year, however, this development is not linked to the pandemic.
- The implementation of measures to contain the pandemic has involved a clear, yet temporary, decline in the utilisation of outpatient general practitioner and specialist services.
- The proportion of people receiving household assistance has remained broadly constant over the observation period.

## Appendix

**Annex Table 1:** Description of the regression models for the calculation of the adjusted proportions/mean values Source: Own table

Independent control variables used in all estimation models: federal state (bula [Baden-Württemberg, …, Thuringia]), age (agegrp [15 to 29 years, 30 to 44 years, 45 to 64 years, 65 to 79 years, ≥ 80 years]), sex (sex [male, female]), CASMIN educational classification (edu [low, medium, high]), and the interactions between age, sex and CASMIN educational classification:
**Variablen ≙ bula + agegrp + sex + edu + agegrp · sex + agegrp · edu + sex · edu**
Monthly adjusted proportions or averages were calculated by adding the interview month (intmo [April 2019, …, September 2020]) as a categorical variable to the basic variables:
**Model 1: health indicator = variables + intmo**
The polynomial of degree four of the interview week (intwo [2019: calendar week 14 to 2020: calendar week 36]) was used to represent a smoothed temporal course:
**Model 2: health indicator = variables + intwo + intwo^2^ + intwo^3^ + intwo^4^**
A binary variable (period [period 2019, period 2020]) has been added to the model estimate for comparing the periods of the years 2019 and 2020 to distinguish the periods:
**Model 3: health indicator = variables + period**

**Annex Table 2:** Comparison of adjusted proportions and mean values between 2019 and 2020 Source: GEDA 2019/2020-EHIS

Indicator	Proportion/average with 95% CI Period 2019 ^1^	Proportion/average with 95% CI Period 2020 ^1^	Number of cases (GEDA 2019/2020-EHIS overall)
Utilisation: general practitioners (%)	38.4 (35.9–41.0)	**29.7** (27.5–31.9)	22,934
Utilisation: Specialist doctors (%)	30.0 (27.6–32.5)	**17.7** (16.0–19.3)	22,892
Mental health: concentration difficulties (%)	21.9 (19.6–24.1)	**18.1** (16.1–20.1)	22,958
Mental health: tiredness, little energy (%)	64.0 (61.5–66.4)	**50.7** (48.3–53.1)	22,959
Mental health: PHQ-8, depressive symptoms (%)	8.3 (6.7 –10.0)	6.6 (5.2–7.9)	22,550
Assistance received (%)^2^	56.5 (50.4–62.5)	61.8 (55.9–67.6)	3,794
Lack of support (%)^2^	26.2 (20.4–32.1)	29.1 (23.1–35.1)	3,782
Assistance provided (%)	20.7 (18.6–22.7)	22.0 (20.1–23.9)	22,979
Tobacco smoking (%)	32.6 (30.2–35.1)	**28.1** (25.9–30.4)	22,991
Daily exposure to passive smoking (%)	5.2 (3.7–6.7)	4.6 (3.2–6.0)	18,089
Body Mass Index (mean value)	25.9 (25.8–26.1)	**26.4** (26.2–26.6)	22,696
Body weight (mean value)	77.1 (76.5–77.6)	**78.2** (77.6–78.9)	22,724

^1^ Reference period for all indicators calendar weeks 15 to 26, for Body Mass Index 15 to 35

^2^ Only participants aged > 55 years

Bold = p-value < 0.05, 95% CI = 95% confidence interval, PHQ-8 = 8-Item Patient Health Questionnaire
