# Corrigendum: Developments in the health situation in Germany during the initial stage of the COVID-19 pandemic for selected indicators of GEDA 2019/2020-EHIS

**DOI:** 10.25646/8384

**Published:** 2021-04-23

**Authors:** Stefan Damerow, Alexander Rommel, Franziska Prütz, Ann-Kristin Beyer, Ulfert Hapke, Anja Schienkiewitz, Anne Starker, Almut Richter, Jens Baumert, Judith Fuchs, Beate Gaertner, Stephan Müters, Johannes Lemcke, Jennifer Allen

Damerow S, Rommel A, Prütz F et al. (2020)

Journal of Health Monitoring 5(4): 3 – 20.

DOI 10.25646/7172

An earlier version of this article gave an incorrect figure on page 5: ‘Based on the standards of the American Association for Public Opinion Research (AAPOR), the response rate was 22.0% (RR3)[15].’

The correct sentence reads: ‘Based on the standards of the American Association for Public Opinion Research (AAPOR), the response rate was 21.6% (RR3)[15].’

The wording of the article in issue 4/2020 was corrected accordingly.

The corrected version of the article is available at DOI 10.25646/7172.2

